# New substitute name for the genus *Poliocoris* Slater, 1994 (Hemiptera, Heteroptera, Rhyparochromidae)

**DOI:** 10.3897/zookeys.478.9063

**Published:** 2015-01-28

**Authors:** Dao-Wei Zhang, Jing Chen

**Affiliations:** 1 Key Laboratory of Regional Characteristic for Conservation and Utilization of Zoology Resource in Chishui River Basin; School of life sciences, Zunyi Normal College, Zunyi, Guizhou, P. R. China, 563002; 2 Zunyi Medical College, Zunyi, Guizhou, P. R. China, 563009

**Keywords:** Hemiptera, Heteroptera, bugs, homonym, replacement name

## Abstract

*Neopoliocoris* **nom. n.**, a new substitute name is proposed for *Poliocoris* Slater, 1994 (Hemiptera: Heteroptera: Rhyparochromidae), preoccupied by *Poliocoris* Kirkaldy, 1910 (Hemiptera: Heteroptera: Pentatomidae). A new combination, *Neopoliocoris umbrosus* (Slater, 1994), **comb. n.** is proposed for *Poliocoris umbrosus* Slater, 1994.

## Introduction

The purpose of the present paper is to provide a new substitute name for a preoccupied Rhyparochromid name in accordance with the International Code of Zoological Nomenclature (1999). The bug genus *Poliocoris* of the family Rhyparochromidae (Hemiptera: Heteroptera) was established by Slater (1994) for the species *Poliocoris umbrosus* Slater, 1994 from South Africa. We have discovered that the genus name *Poliocoris* is preoccupied and was initially introduced by Kirkaldy (1910) for a stink bug genus of the family Pentatomidae (Hemiptera: Heteroptera), with *Poliocoris amnesis* Kirkaldy, 1910 as its type species from the USA (Florissant). According to Article 60 of the International Code of Zoological Nomenclature, we propose the new substitute name *Neopoliocoris* **nom. n.** for *Poliocoris* Slater, 1994. The resulting nomenclatural changes are summarized below.

## Nomenclatural changes

### Genus Neopoliocoris nom. n.

Taxon classificationAnimaliaHemipteraRhyparochromidae

Poliocoris Slater 1994: 120 (Hemiptera: Heteroptera: Rhyparochromidae). Preoccupied by *Poliocoris*Kirkaldy 1910: 130 (Hemiptera: Heteroptera: Pentatomidae).

#### Type species.

*Poliocoris umbrosus* Slater, 1994.

#### Distribution.

South Africa.

#### Etymology.

From the preexisting name *Poliocoris* and the prefix “Neo-” from the Greek “*neos*”, meaning new; gender masculine.

### Neopoliocoris umbrosus

Taxon classificationAnimaliaHemipteraRhyparochromidae

(Slater, 1994) comb. n.

Poliocoris umbrosus Slater 1994: 120.

## Supplementary Material

XML Treatment for Neopoliocoris

XML Treatment for Neopoliocoris umbrosus
